# Foods and Dietary Patterns That Are Healthy, Low-Cost, and Environmentally Sustainable: A Case Study of Optimization Modeling for New Zealand

**DOI:** 10.1371/journal.pone.0059648

**Published:** 2013-03-27

**Authors:** Nick Wilson, Nhung Nghiem, Cliona Ni Mhurchu, Helen Eyles, Michael G. Baker, Tony Blakely

**Affiliations:** 1 Department of Public Health, University of Otago, Wellington, Wellington South, New Zealand; 2 National Institute for Health Innovation, University of Auckland, Auckland, New Zealand; The Ohio State University, United States of America

## Abstract

**Objective:**

Global health challenges include non-communicable disease burdens, ensuring food security in the context of rising food prices, and environmental constraints around food production, e.g., greenhouse gas [GHG] emissions. We therefore aimed to consider optimized solutions to the mix of food items in daily diets for a developed country population: New Zealand (NZ).

**Methods:**

We conducted scenario development and linear programming to model 16 diets (some with uncertainty). Data inputs included nutrients in foods, food prices, food wastage and food-specific GHG emissions.

**Findings:**

This study identified daily dietary patterns that met key nutrient requirements for as little as a median of NZ$ 3.17 per day (US$ 2.41/d) (95% simulation interval [SI] = NZ$ 2.86 to 3.50/d). Diets that included “more familiar meals” for New Zealanders, increased the cost. The optimized diets also had low GHG emission profiles compared with the estimate for the ‘typical NZ diet’ e.g., 1.62 kg CO_2_e/d for one scenario (95%SI = 1.39 to 1.85 kg CO_2_e) compared with 10.1 kg CO_2_e/d, respectively. All of the optimized low-cost and low-GHG dietary patterns had likely health advantages over the current NZ dietary pattern, i.e., lower cardiovascular disease and cancer risk.

**Conclusions:**

We identified optimal foods and dietary patterns that would lower the risk of non-communicable diseases at low cost and with low greenhouse gas emission profiles. These results could help guide central and local government decisions around which foods to focus policies on. That is which foods are most suitable for: food taxes (additions and exemptions); healthy food vouchers and subsidies; and for increased use by public institutions involved in food preparation.

## Introduction

Improving nutrition is a key component of advancing non-communicable disease (NCD) control internationally, with “salt reduction” and “improved diets and physical activity” identified as two of five proposed priority actions [1]. Dietary risk factors and physical inactivity collectively accounted for 10% of global disability-adjusted life years (DALYs) in 2010, with the most prominent dietary risks being diets low in fruits and those high in sodium [2]. Nutritional risk factors are also likely to be exacerbating health inequalities in some populations. This is the case in New Zealand for the indigenous population of Māori relative to the non-Māori (largely European) population [3].

Related to both nutrition and well-being is “food insecurity” and this is often a problem for populations residing within high-income countries. In New Zealand for example, there is clear evidence that food insecurity is a problem for low-income populations [4], [5], [6], [7] and one that is associated with increased psychological distress [8].

Also of relevance when considering nutrition at the population level, are the increasing concerns regarding the sustainability and impact of food production on the environment. This is particularly in terms of greenhouse gas (GHG) emissions, with some estimates being that 19 to 31% of global GHG emissions are related to food production [9]. Of note is that some measures such as replacing red meat consumption with other foods may reduce such GHG emissions and also yield health benefits [10], [11], [12].

These concerns suggest that identifying dietary patterns that are healthy, low-cost, and associated with low GHG emissions, should be of relatively high research and policy interest. One way to study this complex issue of optimizing diets in the context of multiple constraints is through linear programming. This mathematical technique allows the generation of optimal solutions such as identifying the lowest cost mix of foods that satisfy minimum and maximum nutrient levels. Linear programming has been used for decades for informing nutrition, with numerous recent examples for optimizing diets in healthier directions [13], [14], [15], [16]. But it has also started to be used to help identify diets that are associated with low GHG emissions [17], [18].

Given this background, the aim of this study was to perform optimization analyses around foods and dietary patterns to help inform food policies available to central governments concerned with preventing non-communicable diseases, reducing food insecurity, and increasing the sustainability of food production.

## Methods

Dietary optimization work can begin with common dietary patterns and explore incremental steps towards patterns that are lower in cost and better for nutrition. However, the current New Zealand dietary pattern is a poor point-of-departure for such analysis. This is because it is relatively expensive and unhealthy, particularly in terms of cardiovascular risk, i.e., largely the high saturated fat intake [19] but also the high sodium intake [20]. Given these problems, we took a different “bottom-up” approach. This involved obtaining data on a wide range of individual food items and optimizing towards a diet meeting nutritional requirements from there. Nevertheless, for comparison purposes we also included scenarios with “more familiar meals” for New Zealanders.

### Scenarios

We considered four groupings of dietary scenarios that all meet nutrient requirements as well as being: (i) low-cost; (ii) low in GHGs and low-cost; (iii) that were “relatively healthy diets” with high vegetable intakes – a Mediterranean-style diet and an Asian-style diet (within cost and GHG constraints); and (iv) that included “more familiar meals” that would potentially be more acceptable to most New Zealanders.

The first grouping of scenarios focused on achieving the lowest daily food cost while meeting recommended nutrient levels (Scenarios C1–C4, Table 1). Noting the results for the lowest cost option (C1), we added a requirement for basic meal components, e.g., porridge, and inclusion of rotis (naan/flat bread) which utilize low cost flour. Following from Scenario C2, we added a variant allowing for minimal cooking skills (C3), and then a variant that had relatively high vegetable intake (C4; at ≥75% of the diet for men consuming a Mediterranean-style diet [21]). To increase realism we forced additional variety by applying upper limits to certain low-cost foods that tended to be optimized at high volume on the basis of cost and nutrition alone, e.g., the amount of flour, pasta, oats, sugar, kiwi fruit and peanut butter were limited.

**Table 1:** Specific scenarios used for the optimization modeling with a focus on achieving nutrient levels at lowest cost along with low GHG emissions.

Aim of specific scenario	Additional details on the constraints*
***Minimizing cost while meeting nutritional requirements***	
C1) To minimize food cost (while meeting nutrient requirements).	To achieve all the recommended daily energy and nutrient levels (Table S2 in File S1) while minimizing cost. We also limited added sugar to no more than 60 g/d which is half the usual total intake for NZ males).
C2) As per Scenario C1 but to allow(i) a standard porridge dish; and(ii) the flour to be mixed withvegetable oil to make rotis	We required that the flour be considered in conjunction with the inclusion of vegetable oil (at a 7∶1 ratio of flour to vegetable oil). Two cups of flour (240 g) makes around 8–10 rotis, each 10 cm in diameter. Minimal salt in cooking to fall within the nutrient constraints = 0.5 teaspoon (3 g). (We note various alternatives to using the flour in rotis: e.g., scones, damper, tortillas etc. Versions of Cornish pasties and samosas can also be prepared from flour and vegetables.). We included a standard porridge dish at breakfast of ¼ cup (≥39 g) of whole grain oats and ¾ cup of milk made from ≥25 g skim milk powder (where 1 kg makes 10 liters of milk). Minimal salt used in cooking = 0.25 teaspoon (1.5 g).
C3) As per Scenario C1 but requiring very minimal cooking skills	It was assumed that cooking skills were limited to cooking pasta, canned foods, rice and vegetables. The “food skills constraint” ruled out the cooking of: flour (all types), lentils, semolina, couscous and dried peas.
C4) As per Scenario C1 but withextra fruit and vegetables at lowestpossible cost	The additional requirement was for the diet to reach ≥75% of the average intake of vegetables for men consuming a Mediterranean-style diet [21] (i.e., ≥412 g [549 g for men ×75%]). The maximum level for any particular vegetable was set at 200 g, and we excluded starchy root crops (potatoes, kumara and taro) and juices.
***Minimizing GHGs while meeting nutritional requirements***	
G1) To minimize greenhouse gas(GHG) emissions (while achievingthe nutrient levels).	To keep cost to <NZ$ 5 per day.
G2) As above but for a highercost per day	To keep cost to <NZ$ 9/d.**
G3) As per Scenario G2 but with porridge as a standard feature	As per the <$9/d constraint**, but with the porridge and skim milk (as per Scenario C2) included).
G4) As per G2 but fully vegan	As per the <$9/d constraint**, but with exclusions on milk and other dairy products, eggs, fish and all other meat (i.e., vegan).

* In all scenarios (Table 1 and Table 2) we set daily maximum limits for flour, pasta and oats (each at 240 g), no more than 200 g of any particular vegetable (including fresh, frozen, and canned vegetables), and adjusted added salt to ensure that total sodium intake stayed below maximum recommended levels, as per Table S2 in File S1. All the weights in this table are for prepared ready-to-eat items, with purchased weights assumed to be higher due to inedible portions such as skins and spoiled parts – with these wastage proportions obtained from the USDA database) [57]. A lower limit of 10 g applied to all ingredients except condiments such as sugar and salt, and ingredients that were specified in the recipes, e.g., 8 g wholemeal flour in Scenario NZ-M.).

** We based this on an annual survey (the University of Otago “Food Cost Survey”) where for 2011 the calculated costs for a “basic diet” were $9.29/d for men and $8.71/d for women [41].

The second grouping of scenarios (Scenarios G1–G4, Table 1) focused on achieving the lowest GHG profile while meeting nutrient requirements and within varying budget constraints. For contrast we also included a vegan diet with no animal products.

The third grouping of scenarios (“relatively healthy diets”) considered two traditional dietary patterns with aspects that are likely to be health-promoting and had a high vegetable intake. These were a Mediterranean-style diet [21] and an Asian-style diet. The latter shares the high vegetable intake of the Mediterranean diet but in our scenario excludes the typically high-salt sauces (in Table 2: Scenarios MED, MED-G, ASIAN, ASIAN-G). There are now many systematic reviews favoring the impact of vegetable and fruit consumption on health (preventing various cancers [22], [23], [24]; type 2 diabetes [25]; stroke [26]; coronary heart disease [27]; and cognitive decline and dementia [28]). More specifically, systematic reviews also indicate health benefits of the Mediterranean diet e.g., for preventing cardiovascular disease [29], stroke [30], cancer [31] and the metabolic syndrome [32]. Also of note is that Asian-style diets are of increasing relevance to New Zealand with the growing Asian population in the country. There has also been an increase in the number of restaurants selling Asian food in New Zealand.

**Table 2:** Additional scenarios covering specific dietary patterns and aspects of the New Zealand diet.

Aim of specific scenario	Additional details on the constraints
***“Relatively healthy diets” (Mediterranean and Asian)***	
MED) Mediterranean style dietary features	As per Scenario C1 (achieving all nutrients for minimum cost) but with these components based on dietary data for men consuming a Mediterranean-style diet (EPIC cohort study, median values, second table in: Trichopoulou et al) [21] where: (a) Vegetables: ≥549 g for men (for the vegetables listed in Scenario C4 excluding starchy root crops: potatoes, taro and kumara); (b) Fruit and nuts: ≥363 g; (c) Fish and seafood: ≥24 g; (d) Olive oil: ≥56 g; (e) Salt in cooking = 0.5 teaspoon (3 g). (For simplicity we did not specify other “required” components of the Mediterranean diet relating to: legumes, cereals, dairy products, meat products etc, but these could still be selected as part of the optimization process). Fresh and canned fruit could be included.
MED-G) As above but minimizing GHG emissions	To keep cost to <NZ$ 9/d (as per the level in Table 1). In this Scenario we removed the requirement for any fish and seafood.
ASIAN) Asian style dietary features	As per Scenario C1 (achieving all nutrients for minimum cost) but with these additional components: a mix of vegetables, rice (≥200 g) and vegetable oil for stir-fry cooking (≥1 tablespoon (14 g)). In this scenario only we used a supermarket price for bulk rice of $17.99 for 10 kg ($0.18 per 100 g). For the vegetables we set the total amount at ≥500 g with minimum amounts of: carrots (≥50 g); cabbage (≥50 g); broccoli (≥50 g); onion (≥50 g); and Chinese cabbage [“bok choy”] (≥50 g). The 200 g maximum for any particular vegetable also applied. Minimal salt in cooking = 0.5 teaspoon (3 g).
ASIAN-G) As above but to minimizing GHG emissions	To keep cost to <NZ$ 9/d (as per the level in Table 1).
***“More familiar meals” (for New Zealanders) included***	
NZ-M) “Main meal – mince” plus the standard breakfast and lunch	As per Scenario C1 (achieving all nutrients for minimum cost) but with these included components: Evening meal adapted using the key components of the “Mum’s mince on toast” recipe (NZ Beef and Lamb recipe website): beef mince [≥125 g]; onion (medium) [≥28 g], carrot [≥15 g], any other vegetable [≥40 g], wholemeal flour [≥8 g], 1 slice of bread (as toast) [≥28 g] (but note our analysis excluded condiments/sauces). Minimal salt in cooking = 0.33 teaspoon (2 g). *Plus a “standard breakfast”:* Porridge as per Scenario C2 but with sugar as well (0.5 tablespoon, ≥7 g). Minimal salt in cooking = 0.25 teaspoon (1.5 g). *Plus a “standard lunch”:* (a) Half a cheese sandwich (with a whole sandwich comprising: 2 slices of wholemeal bread ≥56 g [28 g slices ×2]; ≥24 g of mild cheddar cheese [the weight of 2 slices (12 g each) in a *generic brand* cheese slices pack]; ≥10 g of margarine); (b) Half a peanut butter sandwich (with a whole sandwich comprising: 2 slices of wholemeal bread ≥56 g [28 g slices ×2]; ≥25 g of peanut butter]; ≥10 g of margarine); and (c) 1 apple (≥130 g].
NZ-S) “Main meal –sausages” plus thestandard breakfastand lunch	As per Scenario C1 (achieving all nutrients for minimum cost) but with these components: (a) sausages (≥96 g, 2 servings); ≥426 g of peeled potatoes (approximately 2 medium potatoes); and any two other vegetables of at least 100 g per vegetable (excluding the starchy root crops of: potatoes, taro and kumara). (b) Dessert: ice cream (≥66 g [1 serving, 0.5 cups]); “canned peaches” or “canned fruit salad” or “canned apricots” (with syrup/juice) ≥147 g [1 serving]. (See Scenario NZ-M for the standard breakfast and lunch).
NZ-T) “Main meal – fish” (“tuna pasta bake”) plus the standard breakfast and lunch	As per Scenario C1 (achieving all nutrients for minimum cost) but with these components: Canned tuna in spring water: ≥124 g (2 servings, drained weight), pasta [≥118 g, 2 servings dry weight]; ≥120 g [0.5 cup] of canned tomatoes; and at least 100 g of any other prepared vegetable (excluding starchy root crops: potatoes, taro and kumara). Minimal salt in cooking = 0.25 teaspoon (1.5 g). (See Scenario NZ-M for the standard breakfast and lunch).
NZ-P) Main meal with“Pacific theme” plusthestandard breakfastand lunch	As per Scenario C1 (achieving all nutrients for minimum cost) but with these components: Taro (peeled) ≥104 g of taro root (equivalent to 1 cup peeled taro root in cubes); canned tuna in spring water ≥77 g (0.5 cup drained weight); “lite” coconut cream ≥222 [0.75 cups]; onion (peeled) ≥14 g [half a medium onion]; vegetable oil ≥7 g (0.5 tablespoon) (proportions based on the recipe for a “taro and seafood” dish*). Taro leaves not included as these may be less readily available in NZ. Minimal salt in cooking = 0.33 teaspoon (2 g). (See Scenario NZ-M for the standard breakfast and lunch).

* For the “taro and seafood” dish the recipe was from: http://www.fao.org/WAIRdocs/x5425e/x5425e01.htm.

The final grouping of scenarios included “more familiar meals”, but with other aspects of the daily diet optimized to achieve nutritional recommendations (Table 1). The meal selection was informed by the “dietary habits” results from the New Zealand Adult Nutrition Survey (NZANS) [19] and the data on food prices (to achieve relatively low cost meals). For example, we selected the evening meal of “main meal – mince” (Scenario NZ-M, Table 1) involving mince on toast. We also included a low-cost breakfast (porridge) and a low-cost lunch (cheese sandwich and peanut butter sandwich). For these meals we used readily available recipes. For example, for the Pacific theme evening meal we used a recipe from the Food and Agriculture Organization of the United Nations website (http://www.fao.org/WAIRdocs/x5425e/x5425e01.htm) and for the mince meal a recipe on the “NZ Beef and Lamb” website.

For comparison purposes, we also entered into our modeling our best estimate of the typical New Zealand diet (for men). This was estimated using national survey data from the NZANS [19] on the proportional contribution of dietary energy intake for major different food categories (excluding alcohol). To each of these categories we assigned relevant food items in varying proportions (some based on NZANS data) for which we had assembled price and nutrient data (n = 76 food items). This gave a total of 9996 kJ of dietary energy and so we then scaled the results to the 11,450 kJ intake used in the other analyses (Table S3 in File S1).

### Selecting the Food Items to Add to the Models

Given the thousands of food products on the New Zealand market, we needed to simplify the options and did this by initially including only foods used in compiling the country’s Food Price Index (FPI) [33]. The FPI includes 44 commonly purchased food items. To expand the range of low-cost foods we also included foods from: (i) previous work that identified low-cost sources of protein in New Zealand [34]; (ii) unprocessed foods (e.g., lentils and peanuts) commonly found in the “bulk bins” at the supermarket and low-cost canned foods (convenience sample in the capital city, Wellington); (iii) lists of selected foods from a previous nutrition optimization study in France [13]; and (iv) foods not covered above, but which were needed to fit with recipes for the lunch and evening meals in the scenarios including “more familiar meals”, e.g., the starchy vegetable taro for the Pacific theme meal. This process resulted in a total of 76 food items (see Table S1 in File S1). To maximize potential health benefits, we ensured that the final selection included relatively healthier variants e.g., unsalted nuts, wholemeal flour, wholemeal bread, low-salt margarine, low-fat ice cream, and “lite” coconut milk.

### Data Inputs (Food Prices, Nutrients, GHGs)

For most of the food items we used official Food Price Index (FPI) price data (monthly data averaged over multiple stores throughout New Zealand for the 12 months of 2011) [33]. However, where food items were not covered in the FPI, we used online supermarket data (Countdown, January 2012), or the lowest in-store (e.g., bulk bin) prices from New World or Countdown supermarkets (both in Karori, Wellington). We took a conservative approach by ignoring prices on “specials” and set the maximum size for food product pricing at 1.5 kg, i.e., generally avoiding savings from bulk purchase.

Nutrient values for the foods were obtained from the 2012 “New Zealand food composition database” (http://www.foodcomposition.co.nz/foodfiles). Estimated nutrient intakes were adjusted to account for food wastage. As detailed food wastage data are not available for New Zealand, we used values from a large United Kingdom (UK) study on food wastage (the WRAP study) [35].

Data available on the GHG profiles of New Zealand foods cover a limited number of foods [36]. Therefore we assumed that the far more comprehensive UK data applied to New Zealand [37], albeit with some approximations for food products not specifically covered. Nevertheless, we conducted a sensitivity analysis that built on available comparison data for the GHGs from food and livestock production between the two countries [36], [38] (see Table S4 and Table S5 in File S1).

### Nutrient Constraints

We focused on meeting average requirements for key macronutrients and micronutrients included in the NZANS [19]. For simplicity, we included only one of the B vitamins, thiamine (for which intakes are inadequate for some populations in the New Zealand setting [19]). The “estimated average requirements” (EARs) of nutrients set for Australia and New Zealand were used along with upper limits for certain nutrients i.e., sodium and vitamin A (Table S2 in File S1) [15]. We took a conservative approach by modeling nutrient requirements for men only. However, the models are still relevant for women because nutrient requirements for women are the same or less than those for men with the exception of iron. To address this difference, we included a constraint for iron such that the EAR value for women (8 mg/day) was used rather than the value for men (6 mg/day) (Table S2 in File S1).

### Approaches to Uncertainty and Heterogeneity

In addition to considering a wide range of scenarios, we explored sources of variability in our estimates. For uncertainty in food prices we generally used the variation in the monthly prices (from the FPI data, fitting to gamma distributions). For non-FPI foods we applied the same patterns observed for the FPI foods in the same food category, e.g., from the median values of the “fresh fruit and vegetable” grouping. Nutrient content of foods also varies (e.g., by variety or brand and level of freshness). Therefore we applied a normal distribution to all the nutrient values with a standard deviation (SD) equal to ±5% of the mean value.

There is substantial uncertainty around food wastage including waste arising from how food is stored. For example, it relates to storage at room temperature or use of refrigeration, eating habits (e.g., eating or removing the skin of fruit or some vegetables), and size of food products, e.g., purchase of larger sized items might lead to relatively more waste [39]. To address such uncertainty for the total food waste proportion, we used the SD calculated from the UK food waste study “WRAP study” (Table 44 [35]) to specify a beta distribution (e.g., potatoes 45% wastage, SD = 1.42%, Table S1 in File S1). For the food items where there was no clear match between the WRAP study and our database, we used the median SD of all the matched food items.

To account for population heterogeneity in nutrition, we utilized the distributional data identified in average nutrient requirements for different types of men. This was for differing body sizes and activity levels for Australia and New Zealand (Table S2 in File S1, and applying normal distributions). However, for the target energy intake of 11,450 kJ we derived distributional values from the published survey results, i.e., based on the 95%CIs in the NZANS [19], we assumed a normal distribution with SD = 184.4 kJ.

### Approach to Mathematical Modeling

We used the simplex algorithm to solve these linear programming problems (see Briend et al [40] for a detailed description of the linear programming). Most of the scenarios were modeled in Microsoft Excel 2010 (Excel Solver, Simplex method). However, where there was a high level of complexity with the food combination options (e.g., selections to achieve a certain level of fruit and vegetables) the R programming language was used (version 2.15.0, lpSolve package). When it was possible to use both approaches, we did this to check the programming quality. Finally, we coded the models and ran 2000 iterations for representative scenarios in the R programming language (version 2.15.0).

## Results

### Low-cost Dietary Patterns

For Scenario C1 all energy and other nutrient requirements for New Zealand men were reached via only nine selected foods. These were (in descending order of quantity): wholemeal flour, pasta, dried peas, eggs, sugar, milk powder, carrots, vegetable oil, and kiwifruit (Table S6 in File S1). In total these foods cost just $3.19 per person per day (Table 3). The Scenario C1 diet would also be healthier than the current New Zealand diet in terms of higher: dietary fiber, potassium, iron, zinc, thiamine, and vitamin E. Similarly it would be healthier in terms of lower: total sugars, saturated fat, and sodium (see Table S2 in File S1, and Table 3).

**Table 3:** Daily costs, emissions of greenhouse gases and nutrient intakes for the different dietary scenarios.

Scenario/Nutrients	C1	C2	C3	C4	G1	G2	G3	G4	ASIAN	ASIAN-G	MED	MED-G	NZ-M	NZ-S	NZ-T	NZ-P
Emissions (kg CO_2_e)*	2.72	2.64	2.2	4.33	**1.67**	**1.31**	**1.56**	**1.9**	4.03	**3.29**	4.68	**2.17**	5.25	4.54	4.24	5.98
Price (<5 NZ$ )*	**3.19**	**3.23**	**4.06**	**3.93**	4.99	6.15	6.83	7	**4.95**	8.3	**5.64**	8.99	**6.22**	**6.71**	**6.14**	**6.75**
Fruit and vegetables (g)	63	64	73	412	80	70	57	57	500	500	912	799	249	477	383	187
Energy (≥11,450 kJ)	11,450	11,450	11,450	11,450	11,450	11,450	11,450	12,879	11,723	11,450	11,788	11,450	12,650	11,450	11,450	11,450
Saturated fatty acids (≤30 g)	6	7	15	8	18	30	30	14	5	25	13	30	20	15	10	26
Polyunsaturated fatty acids (≥13.1 g)	14	17	27	17	83	97	101	76	13	84	14	85	15	13	15	13
Protein (≥52 g)	124	123	124	121	98	119	109	111	109	94	100	88	133	107	139	118
Dietary fiber (≥30 g)	51	53	44	54	54	48	44	65	57	46	57	50	64	48	62	59
**Minerals (selected)**																
Sodium (≤2,300 mg)	475	2,171	332	550	237#	504	812	1,887	1,523	1,330	1,670	1,398	2,300	2,292	2,300	2,300
Total sugars (g)	90	56	32	93	22	11	29	27	43	41	125	103	45	92	45	44
Potassium (≥3,800 mg)	3,800	3,800	3,800	3,800	3,800	3,800	3,800	3,800	3,800	3,800	3,800	4,607	3,800	5,052	3,800	3,800
Calcium (≥840 mg)	840	840	840	840	840	840	840	840	840	840	840	840	840	843	840	840
Iron (≥8 mg)	23	25	23	25	33	26	21	34	19	18	24	19	31	21	28	25
Zinc (≥12 mg)	18	19	15	18	21	21	19	21	15	15	15	15	24	15	19	21
Selenium (≥60 µg)	60	60	60	60	90	148	122	141	60	123	60	106	60	60	101	75
**Vitamins (selected)**																
Vitamin A (≥625 and ≤3,000 µg RE)	625	625	625	625	625	625	625	625	1,700	808	625	2,149	625	1,385	625	625
Thiamine (≥1 mg)	2	2.3	2	2.4	6	7	6.8	7.1	2	5.8	2.1	5.8	3	2.3	2.7	2.3
Vitamin C (≥30 mg)	30	30	30	59	30	30	30	47	118	211	94	153	34	86	44	35
Vitamin D (mcg)	1	1	1	1	0	8	0	1	0	2	3	0	2	2	7	5
Vitamin E (≥10 mg)	11	12	12	13	78	98	101	83	11	90	14	87	11	13	10	11
**Calculated ratios**																
Poly. fats/Sat. fats (ratio)**	2.1	2.3	1.7	2.2	4.7	3.2	3.4	5.3	2.8	3.4	1.1	2.8	0.7	0.9	1.4	0.5
Potassium/Sodium (ratio)**	8	1.8	11.5	6.9	16	7.5	4.7	2	2.5	2.9	2.3	3.3	1.7	2.2	1.7	1.7

* The bolded numbers in these rows refers to the “objective function value” in each scenario (i.e., the key value being minimized in the optimization process).

** Ratios of mean (and median and SI), not the mean ratio.

# This value for sodium of 237 mg/d was near the reported physiological requirement for sodium of 184–230 mg/day [58], though higher levels of intake would be required in certain situations (e.g., for men doing outdoor manual work in hot weather).

The daily cost of the Scenario C1 diet increased modestly (by $0.87), with various changes to ensure more suitability for cooking (e.g., including porridge and a dish using the flour and oil – Scenario C2), and ease of preparation (e.g., easy-to-cook foods only – Scenario C3). The uncertainty and heterogeneity analysis relating to Scenario C1 is shown in Table 4. The upper bound of the 95% simulation interval [SI] for the total daily cost was however, still reasonably low at $3.50 (Table 5).

**Table 4:** Simulation intervals of selected foods (with daily weights of foods in g/day) included in the various daily dietary scenarios for the lowest cost diet (C1); a low-cost and low-GHG emissions diet (G1); and C1 and G1 with NZ GHGs values as a result of the optimization process (n = 2000 iterations).

	Lowest cost, Scenario C1				Low GHG, low cost, Scenario G1				G1 with NZ GHGs values			
Food items	Mean	Median	Lower 95%SI	Upper 95%SI	Mean	Median	Lower 95%SI	Upper 95%SI	Mean	Median	Lower 95%SI	Upper 95%SI
**Fruit and vegetables**												
Potatoes	1	0	0	0	11	0	0	164	2	0	0	0
Carrots	39	40	0	69	52	52	23	79	7	0	0	52
Kumara	0	0	0	0	13	0	0	88	1	0	0	0
Kiwifruit, green*	18	19	0	40	14	16	0	39	68	30	0	200
Sultanas	0	0	0	0	11	0	0	143	13	0	0	152
**Cereals and grains**												
Oats (wholegrain)	7	0	0	109	152	240	0	240	43	0	0	240
Flour (wholemeal)	240	240	240	240	14	0	0	214	8	0	0	137
Pasta	240	240	240	240	1	0	0	0	1	0	0	0
White flour	0	0	0	0	39	0	0	231	35	0	0	204
**Pulses, seeds and nuts**												
Peanuts	0	0	0	0	12	0	0	129	10	0	0	112
Dry peas*	148	150	34	240	69	47	0	236	105	106	0	240
Chickpeas – canned	0	0	0	0	24	0	0	231	50	0	0	240
Sunflower seeds	5	0	0	41	145	151	57	210	127	127	51	207
**Dairy products**												
Milk powder*	46	46	31	62	19	22	0	52	25	31	0	58
Milk (whole, homogenized)	0	0	0	0	164	0	0	538	189	0	0	570
**Other foods (including foods used in generating “more familiar meals”)**												
Eggs*	69	73	0	132	0	0	0	0	0	0	0	0
Spreads – Peanut butter	1	0	0	0	24	0	0	100	22	0	0	100
Oil (vegetable)*	29	29	0	60	50	60	0	60	51	60	0	60
Added sugar*	42	60	0	60	0	0	0	0	0	0	0	0
Margarine	1	0	0	31	0	0	0	0	34	36	0	67
**Total food weights (g/day)** **	**906**	**896**	**545**	**1,488**	**820**	**588**	**80**	**2,839**	**809**	**391**	**51**	**2,785**

* For these three scenarios, these results are the ones that were the only ones ***not*** influenced by a small number of values that are outside of the 95%SI.

NB: No fish or meat products were selected.

** Totals include foods contributing less than 10 g/day (mean value in all scenarios) but which were not shown in the table (i.e., cabbage, broccoli, oranges, canned fruit (apricot halves), rice, wheat germ, salted butter, potato crisps, and juice (apple).

**Table 5:** Simulation intervals (addressing uncertainty and heterogeneity) for daily costs, emissions of greenhouse gases and nutrient intakes for the dietary scenarios with for the lowest cost (C1) diet; and a low-cost and low-GHG emissions diet (G1) as a result of the optimization process (n = 2000 iterations).

Scenario:	Lowest cost – Scenario C1 (C1 with NZ GHGs values)						Low GHG and low cost – Scenario G1						Scenario G1 with estimated NZ GHGs values (see *Methods*)				
Nutrients, etc	Mean	Median	Lower 95%SI bound		Upper 95%SI bound		Mean	Median	Lower 95%SI bound		Upper 95%SI bound		Mean	Median	Lower 95%SI bound		Upper 95%SI bound
Emissions (kg CO_2_e)*	2.67 (2.40)	2.66 (2.40)	2.19 (2.00)		3.15 (2.85)		**1.63**	**1.62**	**1.39**		**1.85**		**1.39**	**1.39**	**1.22**		**1.57**
Price (<5 NZ$)*	**3.18**	**3.17**	**2.86**		**3.50**		4.99	4.99	4.99		4.99		4.99	4.99	4.99		4.99
Fruit and vegetables (g)	63	59	0		136		103	68	23		538		99	30	0		468
Energy (≥11,450 kJ)	11,451	11,448	11,092		11,812		11,450	11,448	11,090		11,812		11,450	11,448	11,090		11,812
Saturated fatty acids (≤30 g)	7	7	4		12		22	22	13		31		29	30	23		35
Polyunsaturated fatty acids (≥13.1 g)	16	15	11		28		75	76	51		96		77	77	50		100
Protein (≥52 g)	123	123	102		143		99	100	72		124		99	100	69		124
Dietary fiber (≥30 g)	51	51	41		64		48	49	32		63		41	40	30		56
**Minerals (selected)**																	
Sodium (≤2,300 mg)	467	463		329	634	280		254		152	606	473		465		263	768
Total sugars (g)	73	85		26	99	33		26		16	125	44		34		21	139
Potassium (≥3,800 mg)	3,792	3,799		3,015	4,525	3,795		3,799		3,044	4,525	3,794		3,800		3,021	4,525
Calcium (≥840 mg)	838	837		683	998	838		837		683	998	838		837		683	998
Iron (≥8 mg)	24	24		18	30	27		28		16	36	22		21		15	32
Zinc (≥12 mg)	18	18		15	22	18		18		13	23	16		16		12	21
Selenium (≥60 µg)	60	60		48	72	84		84		55	114	76		75		53	107
**Vitamins (selected)**																	
Vitamin A (≥625 and ≤3,000 µg RE)	626	625		384	868	626		625		384	868	626		625		384	868
Thiamine (≥1 mg)	2.3	2.2		1.6	3.3	5.1		5.1		3.2	6.7	4.4		4.5		2.7	6.1
Vitamin C (≥30 mg)	32	30		17	43	30		30		18	44	67		34		19	187
Vitamin D (mcg)	1	1		0	6	1		0		0	3	7		8		1	12
Vitamin E (≥10 mg)	13	12		10	23	68		69		38	93	69		69		38	98
**Calculated ratios**																	
Poly. fats/Sat. fats (ratio)**	2.3	2.2		2.8	2.4	3.5		3.5		3.9	3.1	2.6		2.6		2.2	2.8
Potassium/Sodium (ratio)**	8.1	8.2		9.2	7.1	13.5		15		20	7.5	8		8.2		11.5	5.9

* The bolded numbers in these rows refers to the objective function value.

** Ratios of mean (and median and SI), not the mean ratio.

A low-cost diet that had a relatively high intake of vegetables (at least 75% of the level consumed by men with a Mediterranean-style diet) and all other food amounts optimized, was still relatively low-cost at $3.93/d per person (Scenario C4). However, the range of foods in Scenario C4 was also limited, i.e., n = 10 food items in total.

### GHG Emissions

The dietary patterns considered above (C1–C4) were associated with outputs of 2.20 to 4.33 kg of CO_2_ equivalents (CO_2_e) per person per day (Table 3). In comparison, the diet with the lowest impact on GHG emissions was Scenario G2 at 1.31 kg of CO_2_ equivalents (CO_2_e) per day. The emissions were slightly higher for Scenario G1 where the daily cost constraint was tighter (at <$5/d vs $9/d for Scenario G2, Table 3). The fully vegan diet (Scenario G4) resulted in slightly higher GHG emissions and was more expensive than the other “low-GHG emission diets” (Scenarios G1– G3). All the preceding Scenarios (C1–C4; G1, G3) were lacto-ovo vegetarian, i.e., including eggs and milk powder.

The uncertainty and heterogeneity analysis relating to Scenario G1 (Table 4 and Table 5) gave a median result of 1.62 kg CO_2_e/d (95%SI = 1.39 to 1.85 kg CO_2_e). When considering our best estimates for New Zealand-specific GHG values for food production, there were only relatively small differences in the results.

### “Relatively Healthy Diets”

The simplified low-cost Mediterranean diet cost $5.64/d, despite including large amounts of fruit and vegetables, and some fish (Scenario MED). However, dietary variety was also limited (n = 14 foods). Eliminating the fish and optimizing for emissions reduction (Scenario MED-G), lowered the emissions associated with this diet from 4.68 to 2.17 kg CO_2_e/d but increased the cost from $5.64 to $8.99/d.

The simplified Asian style diet was relatively inexpensive at $4.95/d (Scenario ASIAN), although it was also of limited dietary variety (only n = 14 foods, and all vegetarian). Optimizing for low-GHG reduced associated GHG levels, but for a relatively higher cost (at $8.30/d, Scenario ASIAN-G).

### “More Familiar Meals” (for NZ)

**Figure 1:**
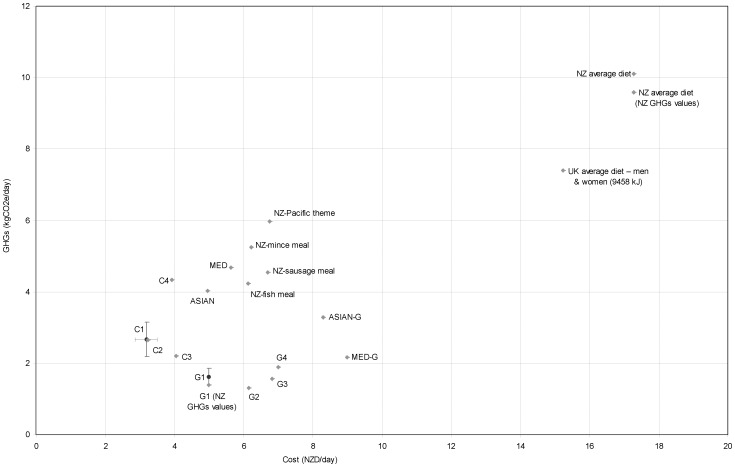
Cost and greenhouse gas (GHG) emissions per day of the various optimized daily dietary scenarios. * *The point estimate for the typical UK diet (at £UK 6.59/d and 7.40 kg CO_2_e/d), came from work by Berners-Lee et al [37], and it is adjusted to NZ$. There are some differences in approach to food wastage by these authors relative to our New Zealand results (i.e. we took a more food-specific approach, albeit also using UK food wastage data). All NZ values are for men consuming 11,450+ kJ and using UK GHGs values for foods unless otherwise indicated. Bars for C1 and G1 indicate 95% simulation intervals (see Table 5).

These diets included familiar meal components but with other aspects optimized (Scenarios NZ-M, NZ-S, NZ-T, NZ-P). They tended to have higher costs at up to $6.75/d (Table 3, Figure 1) compared to the more fully optimized ones, e.g., Scenario C1. However, the cost was still below an estimate for the typical New Zealand diet overall of $17.29/d (Figure 1). These diets also tended to have the highest GHG profile of up to 5.98 kg CO_2_e per/d (for Scenario NZ-P, see Figure 1, Table 3). However, emissions were still far below an estimate for the typical New Zealand diet overall of 10.1 kg CO_2_e/d. While these diets have more elements of likely acceptability for many New Zealanders, the number of different food items was still not particularly large, i.e., a maximum of n = 19 items in Scenario NZ-S.

## Discussion

### Main Findings and Interpretation

This study was able to identify foods and dietary patterns that addressed nutritional requirements for New Zealand adults for as low as NZ$ 3.17/day (US$ 2.41/d). Furthermore, all these diets were likely to be much healthier than the current typical New Zealand dietary pattern in terms of preventing non-communicable diseases. However, increasing the dietary variety and likely acceptability of the diets (via “more familiar meals”) did increase daily cost when optimized for both low-cost and low-GHG emissions. Nevertheless, of the 16 scenarios modeled with some aspect of optimization, only two cost more than $7/d and all were less than half the estimated cost of the current average diet for New Zealand (Figure 1).

These low prices are similar to those for nutritionally optimized daily diets in France at <1.50 €/d [13]. This previous work also found that for diets that were more similar to the typical French pattern, the cost was higher (at 3.40 €/d for men). Survey work in New Zealand has examined “food baskets” of more typically consumed foods and a greater variety of foods e.g., indicating a cost of $65 per week or $9.29/d for a man to eat a “basic” diet [41]. Another New Zealand study reported weekly costs of “regular” and “healthy” food baskets which equated to $12.89/d and $13.80/d respectively per person [42]. It seems that as the modeled optimized diets become more typical of current diets and more varied, they also tend to become more expensive.

From the perspective of cardiovascular disease prevention, there are particular advantages of the low-cost and low-GHG optimized dietary patterns we have described (when compared to the more typical New Zealand dietary pattern, see Table S2 in File S1). These benefits include: the higher ratio of polyunsaturated to saturated fat intake [43], less saturated fat from meat [44], the lower sodium intake [45], and even the higher potassium intake (independent of sodium) [46]. Similarly, the high vegetable diets (Scenarios: C4, MED, ASIAN) would probably be superior to the current New Zealand diet in terms of preventing various non-communicable diseases (as per the systematic reviews cited in the *Methods*). Also, given the high fiber intake (especially from whole grains), there would probably be additional colon cancer risk reduction benefits [47] of these optimized diets. These type of health benefits have been suggested by others who have recently modeled “environmentally sustainable dietary scenarios”, including estimating the “deaths delayed or averted per year” [11].

Our analyses also suggest that while low-cost and low-GHG diets are generally complementary, there is still a modest trade-off between increased daily food cost and consuming food associated with lower GHG emissions. This trade-off is partly because any reduction in higher GHG foods (such as eggs and milk) pushes the optimized food selection in Scenarios G1–G4 towards more expensive alternative foods containing micro-nutrients such as calcium. Other work has similarly reported that milk is a relatively efficient beverage for nutrient provision [48], including in terms of nutrients per GHGs generated [49].

### Study Strengths and Weaknesses

This study was able to integrate considerations of: nutrients, food cost, food wastage and GHG emission profiles from foods. It used both a wide range of scenarios and also simulation analysis. It was more able to generate multiple low-cost healthy diets and ones with low-GHG profiles by attending less to food acceptability than have other such studies, e.g., for the UK [17] and France [18]. While food acceptability issues are important [50], our study was still able to include some scenarios with familiar meal components for the New Zealand population. Also, many of the basic foods that were optimized are used widely around the world, e.g., flour, pasta, oats, carrots and peas.

Yet a limitation of this study was that the food-specific GHG emissions data were from the UK and may differ to various extents from those for New Zealand. Nevertheless, the sensitivity analysis using estimated values for New Zealand did not suggest a major impact on study findings, e.g., a reduction from 1.62 to 1.39 kg CO_2_e for Scenario G1, Table 5. Nonetheless, the GHG emissions data are likely to be underestimates for both countries as they lack consideration of some differential aspects, including: (i) the land use required for food production (which if considered against the counterfactual of land being used for carbon sequestering forest, could substantially increase GHG values for meat and dairy from grazing animals as noted by others [37]); (ii) the emissions from transport of food from shop-to-home; and (iii) the emissions from food being refrigerated and prepared via heating or cooking in the home. Of these, the local shop-to-home transport emissions might possibly be lower in the UK where there is a higher population density and less urban sprawl than New Zealand. Even so New Zealand is also a highly urbanized country with a high density of food retail outlets, especially large supermarkets. In addition, home refrigeration and cooking related GHG emissions would probably be lower in New Zealand, since the majority of national electricity production is non-carbon based, i.e., from hydro, geothermal and wind sources. All these issues highlight the need for more work on individual country estimates of food-specific GHG profiles that capture all the emissions from “farm to fork”, as well as other environmental externalities. These externalities include impacts on biodiversity, water depletion, waterway pollution with pathogens from livestock, and the addition of excess nitrogen in the environment [51].

### Possible Policy Implications

These results could help guide central and local government policies to ensure that optimized foods (healthy, low-cost, and low GHG profiles) are exempt from saturated fat taxes or other taxes on less healthy foods. For example, rather than taxing vegetable oils, as per a former Danish saturated fat tax [52], it seems desirable that such oils are exempted from taxes. While there is an international trend against exemptions from VAT-like taxes [53], countries with such exemptions could ensure that they applied to the “optimized” foods identified by this type of modeling.

Other policy implications include the identification of foods that could be promoted by the health sector and environmental agencies (ideally together) e.g., by food labeling regulations. Such prioritized foods and dietary patterns could be considered in meal design for school lunch programs and for other meals by public institutions, e.g., hospitals, retirement homes, and prisons, etc. There are already several organisations attempting to reduce their GHG footprints, such as the National Health Service in the UK [54]. Such organisations could also commission celebrity chefs and televised cooking shows to demonstrate ways of preparing healthy, low-cost, and sustainable meals using the identified optimized foods. Some celebrity chefs such as Jamie Oliver in the UK already have a track record for promoting healthier foods. At the more basic level there is a need for the education sector to enhance basic food preparation and cooking skills of young people, to facilitate preparation of the low-cost items identified in this optimization work, e.g., porridge, rotis, and stir-fried vegetables.

This work could also inform government policy around a minimum income for healthy living [55], [56], and help to identify the best foods for subsidies or voucher programs to assist families at risk of food insecurity. For example, in the New Zealand setting, vouchers for foods from supermarkets could particularly favor the top foods in Scenario C1 (e.g., flour, pasta, dry peas, eggs, milk powder, carrots, vegetable oil, and kiwifruit). Similarly, vouchers for use at farmers’ markets could particularly be for foods identified in Scenarios C1 and G1 e.g., carrots, kiwifruit, and potatoes. However, this process of identifying optimal foods for vouchers would ideally need to consider such factors as cultural appropriateness and the need to avoid stigmatization, e.g., by discretely building the discounting mechanism into electronic smart cards. Nevertheless, it is possibly best for societies to avoid such targeted approaches and for governments to focus on fiscal and other policies that shift dietary patterns *for the whole population* towards healthier, low-cost, and low-GHG food options. This would ensure providing direct health and economic benefits to all and also wider benefits for protecting the global environment.

## Supporting Information

File S1 **Contains Tables S1–S6.**
(DOC)
