# Supplementary material for: Foods and Dietary Patterns That Are Healthy, Low-Cost, and Environmentally Sustainable: A Case Study of Optimization Modeling for New Zealand
Source: PLoS One. 2013 Mar 27;8(3):e59648. doi: 10.1371/journal.pone.0059648 (PMC3609827; doi:10.1371/journal.pone.0059648)
Supplement: File S1 — Contains Tables S1–S6. (DOC) [file pone.0059648.s001.doc]

**Supporting Information: Additional Methods and Results**

**Table S1. Food products included in the optimization modelling and related input data (GHG emissions, prices and wastage factors).**

| **Foods** | **Details of the selected food and source of price data (ordered by increasing price per 100g in each category)** | **Emissions – (kg CO_2_e/ 100g)*** | **Price ($) per 100g** | **SD – Price ($) per 100g** | **Total wastage % (UK data)**** | **SD – Total wastage %** |
| --- | --- | --- | --- | --- | --- | --- |
| **Fruit and vegetables** | |  |  |  |  |  |
| Potatoes | [Food Price Index (FPI) 2011] uses supermarket and greengrocer (fresh, raw) | 0.04 | 0.17 | 0.04 | 45 | 1.42 |
| Cabbage | [FPI 2011] uses supermarket and greengrocer (fresh, raw) | 0.07 | 0.19 | 0.08 | 46 | 1.98 |
| Carrots | [FPI 2011] uses supermarket and greengrocer (fresh, raw) | 0.07 | 0.22 | 0.03 | 46 | 1.86 |
| Canned vegetables –tomatoes | [Countdown – online] | 0.45 | 0.24 | 0.01 | 14 | 1.18 |
| Chinese cabbage | Also called: “bok choy”, “pak choy”. Price based on: our seasonal adjustment of the Countdown online price (and weight data from purchased sample) | 0.07 | 0.25 | 0.06 | 46 | 1.98 |
| Canned fruit – fruit salad | Juice included. [Countdown – online] | 0.18 | 0.26 | 0.01 | 16 | 1.26 |
| Apples | [FPI 2011] uses supermarket and greengrocer (fresh, raw) | 0.06 | 0.26 | 0.08 | 46 | 2.09 |
| Canned fruit – apricot halves | Juice included. [Countdown – online] | 0.18 | 0.27 | 0.01 | 16 | 1.26 |
| Frozen peas | [FPI 2011] | 0.35 | 0.29 | 0.01 | 14 | 1.37 |
| Kiwifruit, green | [FPI 2011] | 0.06 | 0.34 | 0.29 | 40 | 1.84 |
| Kumara | [Countdown – online] | 0.07 | 0.38 | 0.08 | 46 | 2.09 |
| Bananas | [FPI 2011] The edible proportion of the weight = 64% (USDA) | 0.06 | 0.42 | 0.07 | 43 | 1.39 |
| Canned fruit – peaches | [FPI 2011] (juice included) | 0.19 | 0.42 | 0.03 | 16 | 1.23 |
| Oranges | [FPI 2011] The edible proportion of the weight = 68% (USDA) | 0.06 | 0.46 | 0.03 | 40 | 1.67 |
| Onions | [Countdown – online] fresh, raw | 0.07 | 0.46 | 0.10 | 46 | 1.73 |
| Taro | [Countdown – online] (white) | 0.07 | 0.50 | 0.11 | 46 | 2.14 |
| Lettuce | [FPI 2011] | 0.07 | 0.52 | 0.51 | 55 | 2.03 |
| Sultanas | [FPI 2011] | 0.18 | 0.57 | 0.02 | 16 | 1.26 |
| Broccoli | [FPI 2011] fresh, raw | 0.07 | 0.59 | 0.29 | 46 | 2.58 |
| Tomatoes | [FPI 2011] | 0.61 | 0.65 | 0.56 | 46 | 2.09 |
| Raisins | [Countdown – online] | 0.18 | 0.79 | 0.03 | 16 | 1.26 |
| Mushrooms | [FPI 2011] | 0.95 | 1.07 | 0.08 | 46 | 2.35 |
| **Cereals and grains** | |  |  |  |  |  |
| Flour – white | [FPI 2011] | 0.13 | 0.14 | 0.01 | 16 | 1.33 |
| Flour – wholemeal | Wholemeal [Countdown – Karori] (same as white flour prices in the FPI 2011) | 0.17 | 0.14 | 0.01 | 16 | 1.33 |
| Pasta | *Generic brand* dry spaghetti [Countdown – online] | 0.17 | 0.21 | 0.01 | 16 | 1.47 |
| Rice | Long grain, white [FPI 2011] | 0.66 | 0.25 | 0.01 | 16 | 1.33 |
| Bread – white | [FPI 2011] | 0.13 | 0.29 | 0.01 | 36 | 1.27 |
| Oats (wholegrain) | *Generic brand* Quick oats, 100% wholegrain [Countdown – store] | 0.17 | 0.32 | 0.01 | 16 | 1.50 |
| Semolina | [Countdown – online] | 0.17 | 0.38 | 0.02 | 25 | 1.58 |
| Bread – wholemeal | *Branded* wholemeal bread [Countdown – online] | 0.13 | 0.40 | 0.02 | 36 | 1.70 |
| Spaghetti (canned) | [FPI 2011] | 0.60 | 0.40 | 0.02 | 16 | 1.26 |
| Breakfast biscuits – “Weetbix” | [FPI 2011] | 0.17 | 0.55 | 0.02 | 16 | 1.44 |
| Pop corn | [New World bulk bin] | 0.17 | 0.79 | 0.04 | 25 | 1.84 |
| Wheat germ | [Countdown – online] | 0.17 | 0.92 | 0.04 | 25 | 1.84 |
| Couscous | [New World bulk bin] | 0.17 | 0.99 | 0.05 | 25 | 1.84 |
| **Pulses, seeds and nuts** | |  |  |  |  |  |
| Dry peas | Split peas (green) [Countdown – online] | 0.17 | 0.34 | 0.01 | 14 | 1.37 |
| Chickpeas | Canned [Countdown – online] | 0.17 | 0.60 | 0.02 | 14 | 1.37 |
| Canned lentils | Canned lentils, low-salt (drained weight, Countdown) | 0.45 | 0.65 | 0.03 | 14 | 1.18 |
| Lentils (dried) | Brown or split red lentils [New World bulk bin] | 0.17 | 0.99 | 0.04 | 14 | 1.37 |
| Peanuts | Peanuts (raw, blanched) [New World bulk bin] | 0.18 | 1.09 | 0.05 | 16 | 1.47 |
| Sunflower seeds | Hulled seeds [New World bulk bin] | 0.18 | 1.29 | 0.05 | 16 | 1.47 |
| **Fish and meat** | |  |  |  |  |  |
| Luncheon sausage | Luncheon sliced pork [Countdown – online] | 1.10 | 0.59 | 0.03 | 23 | 1.40 |
| Sausages | [FPI 2011] mix of meats | 0.87 | 0.86 | 0.04 | 23 | 1.40 |
| Beef mince | [FPI 2011] | 1.66 | 1.19 | 0.05 | 23 | 1.63 |
| Sardines | [Countdown] in spring water (with price based on drained weight) | 0.57 | 1.22 | 0.06 | 23 | 1.55 |
| Tuna | [FPI 2011] (canned, supermarket only) Price based on drained weight for generic brand tuna in spring water | 0.57 | 1.30 | 0.09 | 23 | 1.55 |
| Beef, blade steak | [FPI 2011] | 1.66 | 1.42 | 0.06 | 23 | 1.63 |
| Lamb, neck chops | [FPI 2011] | 1.66 | 1.55 | 0.11 | 23 | 1.63 |
| Pork mince | [Countdown – online] | 1.66 | 1.60 | 0.07 | 23 | 1.40 |
| Canned ham | [Countdown – online] | 1.0 | 1.66 | 0.08 | 23 | 1.52 |
| Canned sheep meat | [Countdown – online] | 1.0 | 1.67 | 0.08 | 23 | 1.52 |
| Pork, loin chops | [FPI 2011] | 1.66 | 1.68 | 0.05 | 23 | 1.63 |
| Pie, steak | [FPI 2011] | 0.7 | 1.96 | 0.03 | 35 | 1.72 |
| Canned chicken | [Countdown – online] | 1.0 | 2.11 | 0.12 | 38 | 1.95 |
| Pork, bacon | [FPI 2011] | 0.91 | 2.16 | 0.06 | 23 | 1.40 |
| Beef, sirloin steak | [FPI 2011] | 1.66 | 2.52 | 0.13 | 23 | 1.63 |
| **Dairy products** |  |  |  |  |  |  |
| Milk (whole, homogenized) | [FPI 2011] | 0.13 | 0.18 | 0.00 | 7 | 0.49 |
| Ice cream | [Countdown – online] – vanilla, low fat | 0.39 | 0.30 | 0.01 | 9 | 0.95 |
| Yoghurt | [FPI 2011] | 0.2 | 0.60 | 0.02 | 9 | 0.90 |
| Butter, salted | [FPI 2011] | 0.35 | 0.87 | 0.05 | 9 | 0.95 |
| Milk powder | *Generic brand* skim milk powder [Countdown – online] | 1.30 | 1.00 | 0.04 | 7 | 0.77 |
| Cheese | Mild cheddar [FPI 2011] | 1.50 | 1.02 | 0.06 | 9 | 0.87 |
| **Other foods (including foods to improve fit with cultural patterns)** | | | | | | |
| Soft drink | [FPI 2011] | 0.19 | 0.16 | 0.01 | 9 | 1.06 |
| Sugar | White sugar [FPI 2011] | 0.33 | 0.20 | 0.01 | 16 | 1.30 |
| Salt, table | [Countdown online] | 0.33 | 0.24 | 0.01 | 35 | 1.92 |
| Juice, crisp apple | [FPI 2011] | 0.19 | 0.31 | 0.14 | 14 | 1.27 |
| Coconut cream (lite) | [Countdown – online] | 0.20 | 0.36 | 0.02 | 25 | 1.58 |
| Oil (vegetable) | Canola or blended vegetable [Countdown – online] | 0.17 | 0.47 | 0.02 | 17 | 1.30 |
| Sauce, tomato | [FPI 2011] | 0.61 | 0.52 | 0.03 | 14 | 1.21 |
| Eggs | Medium sized eggs [FPI 2011] | 0.48 | 0.54 | 0.01 | 9 | 0.76 |
| Spreads – Peanut butter | *Generic brand* peanut butter smooth [Countdown – online] | 0.18 | 0.61 | 0.03 | 16 | 1.26 |
| Margarine | [Countdown – online] Reduced salt version | 0.34 | 0.74 | 0.03 | 17 | 1.30 |
| Olive oil | [Countdown – online] | 0.20 | 1.10 | 0.05 | 17 | 1.30 |
| Potato crisps | [FPI 2011] | 0.26 | 1.17 | 0.19 | 6 | 0.73 |
| Biscuit, chocolate coated | [FPI 2011] | 0.23 | 1.55 | 0.06 | 6 | 0.88 |
| Chocolate, dark | [FPI 2011] | 0.27 | 1.55 | 0.04 | 6 | 0.83 |

SD – Standard deviation.

* These values were taken from UK work [1] albeit with NZ estimates used in a sensitivity analysis (see this Supporting Information and Table 5).

** Given the absence of NZ food wastage data we used data from a large UK study (WRAP study) [2]. This provided wastage data on some specific foods but also for the more general food categories (e.g., “fresh fruit”) as per Tables 51 and 52 in the Report on this study (the WRAP study). We used total wastage in the calculations – so that if there was 50% wastage of a food the effective cost per food nutrient gained was doubled. This total wastage includes both non-edible parts of the food (e.g., banana skins) but also potentially edible food that is not eaten (e.g., if it spoils before preparation or is otherwise discarded). In some cases, the values for total waste had to use the very general category of “staple” at 16% or just use the national average for all food in the UK (at 25%).

**Table S2: Nutrient levels used for targets or constraints with most of these being “estimated average requirements” (EARs)* of nutrients per day for adults (based on values set for Australia and New Zealand [3] unless otherwise stated) with comparisons to the current New Zealand diet.**

| **Nutrient** | **EARs or Other Target or Constraint Values Used in the Modelling**  **([RDI] – for comparison)** | | | | **Comment on constraints etc** | **Self-reported intakes (NZANS) – medians** | |
| --- | --- | --- | --- | --- | --- | --- | --- |
|  | **Men** | **SD (% of EAR) [3]** | **SD (absolute value)** | **Women (shown for comparison only)** |  | **Men** | **Women** |
| Energy (kJ) (using estimated energy requirement (EER) [3], averaged for 4 adult age-groups at the mid-range level of physical activity of 1.7 MJ/d) | 11,450 kJ | 1.6 | 180 | 9060 kJ | Intake must reach or exceed this EER in the modelled scenarios. Of note is that these levels may still be higher than ideal for many male New Zealanders given the high prevalence of overweight and obesity. Furthermore, the reported NZANS energy intakes are likely to be under-estimates (i.e., since reported energy intakes decreased between the 1997 and 2008 surveys but obesity rates substantially increased over the same period). There was evidence of under-reporting of energy intake with the previous (1997) national nutrition survey [4] | 10,380 | 7448 |
| Saturated fatty acids | 10% of energy (30g) (upper limit) | 10g** | 2.75g | 19.8g (upper limit) | Intake must be ***equal/below*** this upper limit. These values are the maximum of 10% of dietary energy from this fat source (as per the NHMRC/MoH Report and using the conversion factor used in the NZANS of 37.7 kJ/g). ***Note:*** current intakes for NZ adults are considered too high. | 36.5g (total fat = 95g) | 25.8g (total fat = 67g) |
| Polyunsaturated fatty acids (current intake in NZ from the NZANS) | 13.1g (current) | 10g** | 1.31g | 9.6g (current) | These current levels must be reached/exceeded. The intake of 13.1g is equivalent to 4.8% of dietary energy. | 13.1g | 9.6g |
| Protein (g) | 52 [64] | 12 | 6.24 | 37 [46] | EARs must be reached/exceeded. | 102 | 71 |
| Dietary fiber (g) [Adequate intake = AI] | 30 (AI) | 10** | 3 | 25 (AI) | AI must be reached/exceeded. ***Note:*** no EAR has been set, current intakes for NZ adults are considered too low. | 22.1 | 17.5 |
| **Minerals (selected)** |  |  |  |  |  |  |  |
| Sodium (mg) (upper level) | 2300 (Upper limit) | 10** | 230 | 2300 (Upper limit) | Upper limit must be ***equal/below*** this level. (The NHMRC/MoH Report actually suggests a target of 1600 mg/d (70 mmol) for men and women). ***Note:*** Intakes for NZ adults are relatively high, e.g. recent estimates for NZ men include 2901 mg/d (and excluding consideration of discretionary salt) [5] and 4013 mg/d (based on spot urine data in a national survey) [6]. | Not reported | Not reported |
| Potassium (adequate intake) (mg) | 3800 (AI) | 10** | 380 | 2800 (AI) | AI must be reached/exceeded. ***Note:*** current intakes for NZ adults are considered too low. The NHMRC/MoH has set no EAR or upper limit. | 3449 | 2757 |
| Calcium (mg) | 840 [1000] | 10 | 84 | 840 [1000] | EARs must be reached/exceeded. | 919 | 745 |
| Iron (mg) | 6 [8] | 18 | 1.08 | 8 [18] | In this case for the modelling we used the EAR value for women (8 mg) rather than the value for men (6 mg). | 13.2 | 9.9 |
| Zinc (mg) | 12 [14] | 10 | 1.2 | 6.5 [8] | As above. | 12.9 | 9.0 |
| Selenium (μg) | 60 [70] | 10 | 6 | 50 [60] | EARs must be reached/exceeded. ***Note:*** current intakes for NZ women are considered too low. | 67.0 | 47.1 |
| **Vitamins (selected)** |  |  |  |  |  |  |  |
| Vitamin A (μg RE) | 625 [900] | 20 | 125 | 500 [700] | EARs must be reached/exceeded. Upper limits are 3000 for both men and women. | 846 | 727 |
| Thiamine (mg) | 1.0 [1.2] | 10 | 0.1 | 0.9 [1.1] | EARs must be reached/exceeded. | 1.6 | 1.1 |
| Vitamin C (mg) | 30 [45] | 21 | 6.3 | 30 [45] | As above. | 99 | 99 |
| Vitamin E (Adequate intake: as alpha-tocopherol equivalents) (mg) | 10 (AI) | 10** | 1 | 7 (AI) | AI must be reached. No EAR has been set. | 11.5 | 9.1 |

* The focus here was on the range for the largest adult groupings. Different values may apply to adolescents and older people e.g., those aged 71+ years. The EAR is defined as “a daily nutrient level estimated to meet the requirements of half of the healthy individuals in a particular life stage and gender group.” In some cases “adequate intake” (AI) was used. This is “the average daily nutrient intake level based on observed or experimentally-determined approximations or estimates of nutrient intake by a group (or groups) of apparently healthy people that are assumed to be adequate. Recommended daily intake (RDI) values are also shown were relevant. The National Health and Medical Research Council/Ministry of Health (NHMRC/MoH) work did not set an EAR for carbohydrate due to limited data.

** No standard deviation (SD) was found, and hence one SD was set at 10% of the EAR.

RDI – Recommended dietary intake [3].

**Table S3: Foods used to generate the modeled “typical NZ diet” for men (by equating energy levels in various food categories from national survey data** [7]**)**

| **Food items** | **Amount of food (g) in the model simulating the typical NZ diet (for men)** | **Emissions (kg CO2e)/d** | **NZ$/d (for the selected amount)** |
| --- | --- | --- | --- |
| **Grains and pasta** |  |  |  |
| Flour – white | 6 | 0.01 | 0.01 |
| Flour – wholemeal | 3 | 0.01 | 0.01 |
| Oats (whole grain) | 14 | 0.03 | 0.05 |
| Pasta | 24 | 0.05 | 0.06 |
| Popcorn, air-popped | 2 | 0.00 | 0.02 |
| Rice, (white) | 25 | 0.20 | 0.08 |
| Semolina | 1 | 0.00 | 0.01 |
| **Breads** |  |  |  |
| Bread – white | 178 | 0.36 | 0.79 |
| Bread – wholemeal | 46 | 0.09 | 0.29 |
| **Breakfast cereals** |  |  |  |
| Breakfast biscuits – “Weetbix” | 36 | 0.07 | 0.24 |
| **Biscuits** |  |  |  |
| Biscuit, chocolate coated | 13 | 0.03 | 0.21 |
| Biscuit, plain | 2 | 0.00 | 0.02 |
| **Milk** |  |  |  |
| Milk (homogenized) | 271 | 0.38 | 0.53 |
| Milk (powder, skim) | 4 | 0.06 | 0.05 |
| **Cheese** |  |  |  |
| Cheese, mild cheddar | 23 | 0.37 | 0.25 |
| **Other diary products** |  |  |  |
| Yoghurt | 224 | 0.49 | 1.47 |
| **Butter and margarine** |  |  |  |
| Butter | 4 | 0.01 | 0.04 |
| Margarine | 13 | 0.05 | 0.11 |
| **Eggs and egg dishes** |  |  |  |
| Eggs | 43 | 0.23 | 0.25 |
| **Beef and veal** |  |  |  |
| Beef, blade steak | 20 | 0.44 | 0.38 |
| Beef, sirloin steak | 15 | 0.33 | 0.49 |
| **Poultry** |  |  |  |
| Chicken, cuts | 61 | 0.98 | 2.06 |
| **Sausages and processed meats** |  |  |  |
| Beef, mince | 22 | 0.47 | 0.34 |
| Luncheon sausage | 10 | 0.15 | 0.08 |
| Pastrami, beef | 1 | 0.02 | 0.04 |
| Pork mince | 43 | 0.92 | 0.88 |
| Sausage | 25 | 0.28 | 0.28 |
| **Pies and pasties** |  |  |  |
| Pie, steak | 40 | 0.43 | 1.20 |
| **Fish and seafood** |  |  |  |
| Sardines (canned) | 12 | 0.09 | 0.19 |
| Tuna (canned) | 53 | 0.39 | 0.89 |
| **Potatoes, kumara and taro** |  |  |  |
| Potatoes | 255 | 0.19 | 0.81 |
| Kumara | 9 | 0.01 | 0.07 |
| Taro | 10 | 0.17 | 0.09 |
| **Vegetables** |  |  |  |
| Broccoli | 18 | 0.02 | 0.20 |
| Cabbage | 62 | 0.08 | 0.22 |
| Carrots | 97 | 0.13 | 0.40 |
| Lettuce | 33 | 0.05 | 0.38 |
| Mushrooms | 41 | 0.72 | 0.81 |
| Onions | 36 | 0.05 | 0.31 |
| Peas (frozen) | 24 | 0.10 | 0.08 |
| Tomatoes (canned) | 87 | 0.45 | 0.24 |
| **Fruit** |  |  |  |
| Apples | 79 | 0.09 | 0.39 |
| Banana | 19 | 0.02 | 0.09 |
| Canned fruit – apricot halves | 6 | 0.01 | 0.02 |
| Canned fruit – fruit salad | 7 | 0.01 | 0.02 |
| Canned fruit – peaches | 7 | 0.02 | 0.04 |
| Canned fruit – pears | 10 | 0.02 | 0.06 |
| Kiwifruit (green) | 29 | 0.03 | 0.16 |
| Oranges | 50 | 0.05 | 0.26 |
| Raisins | 4 | 0.01 | 0.04 |
| Sultanas | 2 | 0.00 | 0.01 |
| **Sugar and sweets** |  |  |  |
| Chocolate, dark | 13 | 0.04 | 0.21 |
| Sugar | 22 | 0.08 | 0.05 |
| **Savoury sauces and condiments** |  |  |  |
| Sauce, tomato | 5 | 0.04 | 0.03 |
| Peanut butter | 1 | 0.00 | 0.00 |
| **Non-alcoholic beverages** |  |  |  |
| Apple juice | 178 | 0.39 | 0.65 |
| Soft drink | 177 | 0.37 | 0.31 |
| **Totals*** | **2517**** | **10.10** | **17.29** |

* Totals are slightly different from that expected from the tabulated values, due to rounding.

** This amount is relatively larger than other results in this study due to the inclusion of various drinks (whole milk, apple juice, soft drinks etc).

**Additional Methods Relating to Estimating GHG Emission Profiles for New Zealand foods**

Specific data on the GHG profiles of New Zealand foods covers a very limited range of foods [8] and so we assumed that the far more comprehensive UK data applied to New Zealand [1], albeit with some approximations for food products not specifically covered. Nevertheless, we conducted a sensitivity analysis that built on available comparison data for the GHGs from food and livestock production between the two countries [8], [9] (see main article). In the text below (and Tables S4 and S5), we give our rationale for the estimates for GHGs for various New Zealand food groups.

As part of the sensitivity analysis we used a standard deviation (SD) of ±10% of the point estimates and we derived these estimates from comparison data for the GHGs from food and livestock production between the two countries [8], [9]. This cross-country comparison gave higher New Zealand-specific GHG values for vegetables (133% of the UK values) due to higher fuel inputs, fertilizer, and pesticide inputs for production in New Zealand. However, New Zealand GHG estimations were lower than the UK for fruit (32% of the UK value based on apple production data), beef and sheep meat (88%), and dairy products (61%). The latter two values reflect a mix of higher methane emissions for pastoral farming in New Zealand but substantially lower carbon dioxide emissions from livestock and dairy farming in New Zealand.

**Table S4. Methods for determining best estimates for the sensitivity analysis around GHG emissions associated with New Zealand foods (relative to the UK values used in the baseline analysis).**

| **Food categories** | **NZ values estimated relative to UK values** (for uncertainty analysis we used SD= (10% of the point estimate) | **Comments** |
| --- | --- | --- |
| Vegetables (grown in NZ) | 133% of the UK values (60 vs 45 kg CO2/tonne) | Based on a NZ vs UK comparison study of onion production.* NZ production involved higher inputs of fuel, fertilizer and pesticides (though emissions associated with buildings and equipment were slightly less). A complexity not considered is that foods may have different storage requirements. For example, in contrast to the UK, NZ onions have been bred for long storage (the cultivar “Pukekohe Longkeeper”) and so need less refrigeration and may involve less wastage from spoilage. |
| Fruit (grown in NZ) & fruit juice made in NZ (apple juice) | 32% of the UK values (60 vs 186 kg CO2/tonne) | Based on a NZ vs UK comparison study of apple production.* NZ production involved substantially lower fuel inputs, and lower fertilizer and pesticide inputs in this comparison study. Potentially this analysis is slightly weighted in favor of the UK in that emissions associated with capital items (buildings and equipment etc), were only available for NZ. |
| Beef (produced in NZ) | 88% of the UK value | We used data indicating that methane is the dominate GHG from beef production at 66% (see Figure 2 in Wirsenius et al [10]) and used the data in Table S5 below (showing higher methane from NZ pastoral beef production). For the remaining GHGs we used the NZ vs UK comparison for sheep meat*, i.e., NZ at 20% of the UK values (563 vs 2849 kg CO_2_/tonne). |
| Sheep meat (lamb), (produced in NZ) | 88% of the UK value | As per the assumptions for beef production. |
| Dairy products (milk, butter, cheese, ice cream, yoghurt) | 61% of the UK value | This was based on methane emissions as this GHG dominates over other GHGs associated with dairy product production (see Figure 2 in Wirsenius et al [10]). In comparison, one study that only considered CO_2_, found a lower value for NZ of 44% of the UK value (1298 vs 2921 kg CO_2_/tonne).* This analysis did not consider palm kernel imports (from Indonesia) used as dairy feedstock in NZ (with such production sometimes associated with deforestation). But FAO data also show increasing palm kernel imports into the UK (data for 2000 to 2006) [11]. |
| Fish | UK values used | For the low-priced fish used in our analysis (tuna from Thailand and imported sardines), we assumed the same values for both countries. |
| Fruit and vegetables imported into NZ (bananas, taro) | UK values used | This applied only to the bananas and taro in our analysis (only a small percentage of other fruit is imported in NZ). |
| Cereals and grains | UK values used | While NZ cereal production is lower per capita (64% of the UK by hectare using FAO data [11]), it is of probably similar nature to the UK. Also transport distances for Australian cereal imports to NZ are not likely to be major components (based on shipping emissions data in Saunders et al [8]). |
| All other foods | UK values used | This grouping includes pulses, seeds, nuts, pork products (luncheon, mince, ham etc), poultry (e.g., canned chicken), eggs, and various processed foods (e.g., soft drinks, spreads, crisps, biscuits, and confectionary). For some imported foods distances for the UK might be less (e.g., olive oil to the UK from Southern Europe and chocolate from Europe), but in other cases NZ might be closer to the exporting country (e.g., products such as “coconut cream” from Pacific islands and peanut butter from Australia). |

* Based on data in Saunders et al [8] albeit excluding the data they used for comparisons based on shipping NZ food products to the UK and for refrigerated storage “to meet the same market window” (i.e., as for apples and onions). Of note is that the Saunders et al analysis did not include methane generation from livestock. The life cycle analysis was also restricted to a “cradle-to-plate” analysis, rather than a full “cradle-to-grave” analysis (i.e., it omitted the disposal and waste management phase of the food production cycle). It also did not include within country travel of the finished food product. Nevertheless, it was relatively thorough and included for example the lower UK emissions associated with shorter shipping distances for Middle East oil to the UK, relative to NZ.

**Table S5. Comparison of “implied emission factors” for methane (CH4) for dairy cattle, beef cattle and sheep (UK vs NZ for 2008, data from a NZ Government Report** [9]**).**

| **Country** | **Dairy cattle**  (kg CH_4_/head/year) | **Beef cattle** (kg  CH_4_/head/year) | **Sheep**  (kg CH_4_/head/year) |
| --- | --- | --- | --- |
| **Methane from enteric fermentation** | | | |
| United Kingdom | 105 | 43 | 4.7 |
| New Zealand | 77 | 57 | 10.9 |
| **Methane from manure management** | | | |
| United Kingdom | 25.79 | 4.18 | 0.11 |
| New Zealand | 3.31 | 0.7 | 0.11 |
| **Totals (additional calculations not in the MFE Report)** | | | |
| United Kingdom | 130.79 | 47.18 | 4.81 |
| New Zealand | 80.31 | 57.7 | 11.01 |
| NZ total as a % of the UK total | 61.4% | 122.3% | 228.9% |

**Additional Results**

**Table S6. Foods (with weights) selected by the optimization process for the various daily dietary scenarios.**

| **Food items** (and specific forms used in the nutrient analysis) | **Total food weights from the optimization process per day (g) (for foods selected in any of the scenarios)** | | | | | | | | | | | | | | | |
| --- | --- | --- | --- | --- | --- | --- | --- | --- | --- | --- | --- | --- | --- | --- | --- | --- |
| **Scenario:** | **C1** | **C2** | **C3** | **C4** | **G1** | **G2** | **G3** | **G4** | **ASIAN** | **ASIAN-G** | **MED** | **MED-G** | **NZ-M** | **NZ-S** | **NZ-T** | **NZ-P** |
| **Fruit and vegetables** |  |  |  |  |  |  |  |  |  |  |  |  |  |  |  |  |
| Potato (Red King, flesh & skin, raw) | 0 | 0 | 121 | 0 | 0 | 57 | 0 | 0 | 0 | 0 | 0 | 0 | 0 | 426 | 0 | 0 |
| Cabbage (white, raw) | 0 | 0 | 0 | 0 | 0 | 0 | 0 | 0 | 200 | 200 | 130 | 200 | 40 | 0 | 0 | 0 |
| Carrot, raw | 39 | 39 | 52 | 21 | 57 | 53 | 57 | 57 | 150 | 50 | 19 | 200 | 41 | 100 | 33 | 43 |
| Canned vegetables – tomatoes diced | 0 | 0 | 0 | 200 | 0 | 0 | 0 | 0 | 0 | 0 | 200 | 0 | 0 | 100 | 120 | 0 |
| Pak choy (Chinese cabbage) | 0 | 0 | 0 | 0 | 0 | 0 | 0 | 0 | 50 | 50 | 0 | 149 | 0 | 0 | 0 | 0 |
| Canned fruit – fruit salad in syrup | 0 | 0 | 0 | 0 | 0 | 0 | 0 | 0 | 0 | 0 | 210 | 0 | 0 | 147 | 0 | 0 |
| Apple, assorted varieties | 0 | 0 | 0 | 0 | 0 | 0 | 0 | 0 | 0 | 0 | 0 | 0 | 130 | 130 | 130 | 130 |
| Canned fruit – apricot halves | 0 | 0 | 0 | 0 | 0 | 0 | 0 | 0 | 0 | 0 | 153 | 0 | 0 | 0 | 0 | 0 |
| Peas (green, frozen, uncooked) | 0 | 0 | 0 | 191 | 0 | 0 | 0 | 0 | 0 | 0 | 200 | 0 | 0 | 0 | 100 | 0 |
| Kiwifruit, green | 24 | 25 | 11 | 0 | 23 | 0 | 0 | 0 | 0 | 0 | 0 | 0 | 10 | 0 | 0 | 0 |
| Kumara | 0 | 0 | 0 | 0 | 0 | 0 | 64 | 125 | 0 | 0 | 0 | 0 | 0 | 0 | 0 | 0 |
| Orange (flesh, fresh) | 0 | 0 | 0 | 0 | 0 | 0 | 0 | 0 | 0 | 0 | 0 | 183 | 0 | 0 | 0 | 0 |
| Onions | 0 | 0 | 0 | 0 | 0 | 0 | 0 | 0 | 50 | 50 | 0 | 0 | 28 | 0 | 0 | 14 |
| Taro | 0 | 0 | 0 | 0 | 0 | 0 | 0 | 0 | 0 | 0 | 0 | 0 | 0 | 0 | 0 | 104 |
| Sultanas | 0 | 0 | 10 | 0 | 0 | 0 | 0 | 0 | 0 | 0 | 0 | 67 | 0 | 0 | 0 | 0 |
| Broccoli | 0 | 0 | 0 | 0 | 0 | 17 | 0 | 0 | 50 | 150 | 0 | 0 | 0 | 0 | 0 | 0 |
| **Cereals and grains** |  |  |  |  |  |  |  |  |  |  |  |  |  |  |  |  |
| Flour, wholemeal | 240 | 240 | 0 | 240 | 0 | 0 | 0 | 0 | 240 | 0 | 240 | 0 | 240 | 162 | 240 | 240 |
| Pasta (egg, dry, uncooked) | 240 | 240 | 240 | 240 | 0 | 0 | 0 | 0 | 240 | 0 | 240 | 0 | 240 | 240 | 212 | 172 |
| Rice (white, polished) | 0 | 0 | 0 | 0 | 0 | 0 | 0 | 0 | 200 | 200 | 0 | 0 | 0 | 0 | 0 | 0 |
| White bread, sliced, pre-packed | 0 | 0 | 0 | 0 | 0 | 0 | 0 | 393 | 0 | 0 | 0 | 0 | 0 | 0 | 0 | 0 |
| Oats, whole grain, raw | 0 | 39 | 240 | 0 | 240 | 81 | 39 | 240 | 0 | 0 | 0 | 0 | 161 | 39 | 115 | 116 |
| Wholemeal bread (sliced & unsliced) | 0 | 0 | 0 | 0 | 0 | 0 | 0 | 0 | 0 | 0 | 0 | 0 | 84 | 56 | 56 | 56 |
| **Pulses, seeds and nuts** |  |  |  |  |  |  |  |  |  |  |  |  |  |  |  |  |
| Peas, split, uncooked | 156 | 140 | 0 | 91 | 96 | 0 | 0 | 0 | 31 | 0 | 0 | 0 | 11 | 0 | 35 | 33 |
| Peanuts, raw | 0 | 0 | 0 | 0 | 0 | 180 | 174 | 0 | 0 | 52 | 0 | 113 | 0 | 0 | 0 | 0 |
| Sunflower seeds (kernel, dried) | 0 | 0 | 10 | 0 | 175 | 210 | 210 | 210 | 13 | 210 | 10 | 189 | 11 | 15 | 0 | 0 |
| **Fish and meat** |  |  |  |  |  |  |  |  |  |  |  |  |  |  |  |  |
| Sausage | 0 | 0 | 0 | 0 | 0 | 0 | 0 | 0 | 0 | 0 | 0 | 0 | 0 | 96 | 0 | 0 |
| Beef mince | 0 | 0 | 0 | 0 | 0 | 0 | 0 | 0 | 0 | 0 | 0 | 0 | 125 | 0 | 0 | 0 |
| Sardines (drained solids, canned) | 0 | 0 | 0 | 0 | 0 | 71 | 0 | 0 | 0 | 0 | 24 | 0 | 0 | 0 | 0 | 0 |
| Tuna (canned in spring water, drained) | 0 | 0 | 0 | 0 | 0 | 0 | 0 | 0 | 0 | 0 | 0 | 0 | 0 | 0 | 124 | 77 |
| **Dairy products** |  |  |  |  |  |  |  |  |  |  |  |  |  |  |  |  |
| Milk (whole homogenized, 3.3% fat) | 0 | 0 | 0 | 0 | 0 | 0 | 23 | 0 | 0 | 327 | 0 | 0 | 0 | 0 | 0 | 0 |
| Ice cream | 0 | 0 | 0 | 0 | 0 | 0 | 0 | 0 | 0 | 0 | 0 | 0 | 0 | 66 | 0 | 0 |
| Powdered milk, skim | 47 | 44 | 37 | 42 | 29 | 0 | 33 | 0 | 39 | 0 | 28 | 24 | 27 | 25 | 31 | 32 |
| Cheese (cheddar, mild) | 0 | 0 | 0 | 0 | 0 | 0 | 0 | 0 | 0 | 0 | 0 | 0 | 12 | 12 | 12 | 12 |
| **Other foods (including foods to improve fit with cultural patterns)** | | | | | | | |  |  |  |  |  |  |  |  |  |
| Sugar | 60 | 27 | 0 | 60 | 0 | 0 | 0 | 0 | 0 | 0 | 0 | 0 | 7 | 7 | 7 | 7 |
| Salt, table | 0.0 | 4.5 | 0.0 | 0.0 | 0.0 | 0.0 | 1.5 | 0.0 | 3.0 | 3.0 | 3.0 | 3.0 | 3.5 | 1.5 | 3.0 | 3.5 |
| Coconut cream | 0 | 0 | 0 | 0 | 0 | 0 | 0 | 0 | 0 | 0 | 0 | 0 | 0 | 0 | 0 | 222 |
| Oil (vegetable, blend) | 27 | 34 | 0 | 36 | 57 | 0 | 20 | 0 | 14 | 19 | 0 | 0 | 0 | 0 | 14 | 11 |
| Egg (whole, raw) | 81 | 80 | 37 | 83 | 0 | 0 | 0 | 0 | 31 | 0 | 27 | 0 | 10 | 12 | 0 | 0 |
| Peanut butter spread | 0 | 0 | 144 | 0 | 0 | 0 | 0 | 0 | 0 | 0 | 0 | 0 | 13 | 13 | 13 | 13 |
| Margarine | 0 | 0 | 0 | 0 | 0 | 0 | 0 | 0 | 0 | 0 | 0 | 0 | 10 | 10 | 10 | 10 |
| Olive oil | 0 | 0 | 0 | 0 | 0 | 0 | 0 | 0 | 0 | 0 | 56 | 56 | 0 | 0 | 0 | 0 |
| **Total food weights consumed per day (g)** | **914** | **914** | **902** | **1,203** | **678** | **669** | **622** | **1,025** | **1,311** | **1,311** | **1,540** | **1,184** | **1,204** | **1,658** | **1,255** | **1,295** |
| **Number of food items** | **9** | **11** | **10** | **10** | **7** | **7** | **9** | **5** | **14** | **11** | **14** | **10** | **19** | **19** | **17** | **18** |

**References**
